# Clinical features of poorly distinguishable HFMD and chickenpox in children: a retrospective analysis

**DOI:** 10.3389/fped.2025.1706776

**Published:** 2025-12-10

**Authors:** Qiuxia Yang, Yiteng Zhang, Jiaxin Duan

**Affiliations:** 1 Department of Laboratory Medicine, West China Second University Hospital, Sichuan University, Chengdu, China; 2 Key Laboratory of Birth Defects and Related Diseases of Women and Children, Sichuan University, Ministry of Education, Chengdu, China

**Keywords:** hand-foot-mouth disease, chickenpox, clinical diagnosis, children, coinfection

## Abstract

Atypical Hand-foot-mouth disease (HFMD), which is caused predominantly by enteroviruses (EVs) shares overlapping cutaneous manifestations with varicella, a condition triggered by varicella-zoster virus (VZV) and further complicates diagnosis. This study systematically characterized the clinical features, hematological profiles, and infection patterns (single, mixed and sequential infection) of pediatric patients with poorly distinguishable EV and VZV infection to improve diagnostic accuracy and optimize clinical management strategies. A retrospective analysis was conducted on the cases that were difficult to distinguish and were confirmed by polymerase chain reaction (PCR) from 2021 to 2025, including demographics, symptoms, hematological profiles, and infection patterns. Among the 85 patients, 33 had a single EV infection and 46 had a single VZV infection, with 4 mixed infections and 2 sequential infections. Compared with patients in the VZV group, those in the EV group were more often associated with local rashes on the limbs and around the mouth, often accompanied by oral vesicles. In contrast, patients with VZV infection usually exhibited pruritic, widespread skin rashes across the body. Virological testing identified CV-A6 as the dominant EV serotype (51.52%). Hematological profiles revealed increased atypical lymphocytes in patients with VZV infection compared with those with EV infection. Patients with mixed infection maybe correlated with more extensive skin lesions. The clinical differentiation of poorly distinguishable EV and VZV infection presents significant challenges, especially in children with mixed infection or atypical features. In the absence of early virological testing, it is hoped that this study can provide some guidance for the diagnosis of HFMD and chickenpox that are difficult to distinguish or have mixed infection.

## Introduction

Enterovirus (EV) and varicella-zoster virus (VZV) are two prominent viral pathogens that commonly affect children worldwide. Hand‒foot‒mouth disease (HFMD), which is classically associated with EV-A71 and Coxsackievirus A16 (CV-A16), manifests as febrile illness accompanied by nonpruritic maculopapular or vesicular eruptions localized on the palmar, plantar, and oral regions (1). However, recent epidemiological studies have highlighted the rise of atypical HFMD variants driven by emerging serotypes such as Coxsackievirus A6 (CV-A6). These strains exhibit a broader anatomical distribution, involving the trunk and limbs, and produce vesiculobullous lesions resembling those of varicella (2–5), thereby complicating clinical differentiation (6).

In contrast, the hallmark centrifugal exanthem progression of chickenpox under VZV infection follows a distinct temporal trajectory: successive waves of pruritic macules, papules, vesicles, and crusted lesions coexist simultaneously within the same anatomical field (7). While virologically divergent, the increasing convergence of cutaneous manifestations in patients with atypical EV infection and modified varicella presentations creates diagnostic ambiguities (8), particularly in endemic regions where both viruses exhibit seasonal cocirculation. These clinical parallels necessitate a critical reevaluation of traditional syndromic classification frameworks.

Conventional laboratory diagnostic methods, such as complete blood count (CBC), often fail to adequately distinguish between EV and VZV infection because of significant overlap in hematological profiles. For example, despite a higher incidence of atypical lymphocytes in VZV patients, this parameter lacks specificity for definitive diagnosis. This diagnostic insufficiency underscores the imperative for molecular confirmation via polymerase chain reaction (PCR) and serotype-specific assays. Notably, CV-A6 has emerged as the predominant EV serotype in severe HFMD cases, underscoring its clinical significance in pediatric populations.

The diagnostic complexity increases further in cases of viral coexistence, which is a relatively rare report but is associated with severe clinical manifestations, including extensive dermatological involvement, prolonged illness duration, and heightened risks of complications such as encephalitis (9, 10). Sequential infection, where one virus precedes the other, present temporal patterns that may reflect potential virus‒virus interactions (11). These interactions, although not fully understood, likely modulate disease severity and immune responses, warranting further investigation.

This investigation systematically compares the clinical and laboratory characteristics of pediatric EV and VZV infection, with a focus on distinguishing atypical HFMD presentations and understanding the impact of mixed infection and sequential infection. By identifying distinguishing markers and analyzing infection patterns, this research seeks to improve diagnostic strategies, inform clinical management and contribute to a broader understanding of virus‒virus interactions.

## Materials and methods

### Study design

Children with HFMD or chickenpox who could not be distinguished at the first visit to West China Second Hospital from January 2021 to July 2025 were retrospectively analyzed. This was subsequently confirmed by RT-PCR testing as HFMD, chickenpox or both. Patients with chronic conditions or immunodeficiency that could affect clinical outcomes were excluded. Patient records were reviewed for clinical presentation, laboratory findings, and virological tests (RT-PCR for EV and VZV) at the first visit. The study protocol conformed to the ethical guidelines of the 1975 Declaration of Helsinki and was approved by the Clinical Research Ethics Committee of West China Second University Hospital, Sichuan University [Medical Research 2024 Ethics Approval (279)]. Owing to the nature of retrospective studies, informed consent from individual patients was waived. Patient data confidentiality was maintained, and all analyses were performed on de-identified data to ensure privacy.

For the purpose of this study, sequential infection was defined as two separate, laboratory-confirmed (by PCR) symptomatic infection with EV and VZV occurring in the same individual, separated by a time interval of at least 14 days. This criterion was established based on the typical incubation and illness periods of both viruses to ensure that the second infection represented a new, independent event after the complete resolution of symptoms (including skin lesions) from the first infection. Mixed infection was defined as the simultaneous detection of both EV and VZV nucleic acids in a sample collected during a single disease episode.

### Data collection

Data collected from medical records included demographic information (age, sex), clinical symptoms (fever, rash characteristics such as location, type, pruritus, oral or pharyngeal lesions, and other associated symptoms), laboratory tests, and virological testing. Laboratory tests focused on complete blood count (CBC), including leukocyte counts and percentages of neutrophils, lymphocytes, and monocytes, with specific attention to the presence of atypical lymphocytes.

### Virological testing

Nucleic acid isolation followed pathogen-specific protocols: EV RNA was extracted via the DAAN GENE Viral RNA Kit (Guangzhou, China), whereas VZV DNA was performed via the Sansure Nucleic Acid Extraction System (Changsha, China). Real-time PCR amplification and quantification were conducted on an ABI 7500 Fast System (Thermo Fisher Scientific, USA) via commercial multiplex assays for detecting and analyzing EVs (universal EVs, EV-A71, CV-A16, CV-A6 and CV-A10) (DAAN GENE, China) and VZV (Sansure, China). The sample results were determined to be positive or negative following the instructions of the respective kits.

### Statistical analysis

The differences in clinical features and laboratory results between the EV group and VZV group were assessed using appropriate statistical tests. Categorical variables were compared using the chi-square test or Fisher's exact test, as appropriate. Continuous variables, which were not normally distributed and the relatively small sample size of the cohort, were compared using the nonparametric Mann–Whitney *U* test. Given the exploratory nature of this study aimed at characterizing clinical features, and considering the modest sample size, no adjustments for multiple comparisons were made. The reported *P* values should therefore be interpreted descriptively and with caution, as they are intended to highlight potential differences rather than to confirm definitive associations. To address this deficiency, we no longer solely rely on the *P* value. Instead, for categorical variables comparisons, we calculated and reported the effect size, such as odds ratio and its 95% confidence interval. Statistical analyses were conducted via GraphPad Prism 8.0 and SPSS software 19.0, and graphical representations were generated via GraphPad Prism 8.0. A value of *P* < 0.05 was considered significant.

## Results

### Overview of the hardly distinguishable cases between HFMD and chickenpox

**Figure 1:**
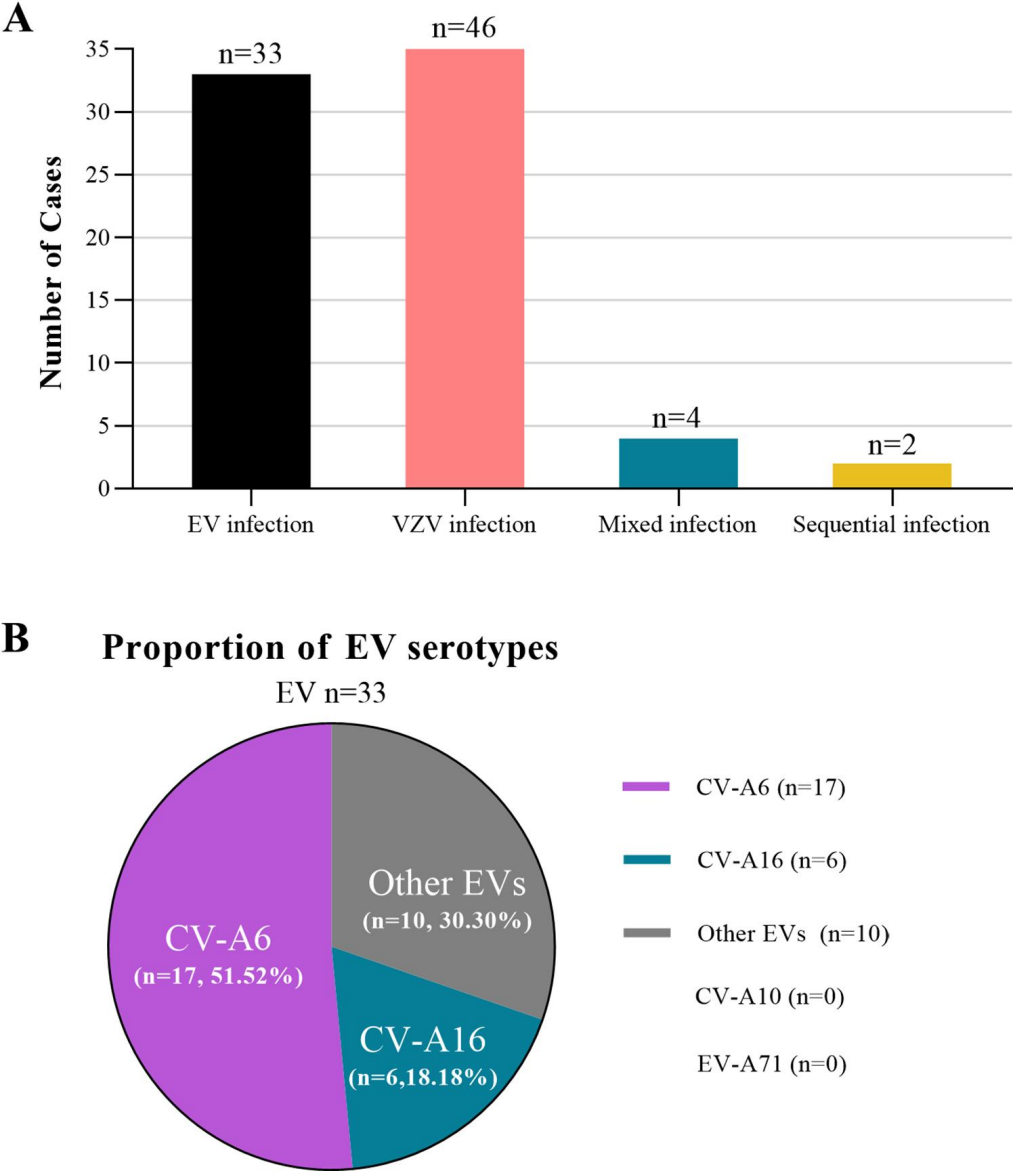
Overview of the hardly distinguishable cases between HFMD and chickenpox. **(A)** The four hardly distinguishable HFMD and chickenpox groups including laboratory-confirmed EV infection, laboratory-confirmed VZV infection, mixed infection and sequential infection with both viruses. **(B)** Proportion of EV serotypes in the EV group.

To analyze the clinical characteristics of pediatric patients with hardly distinguishable HFMD-infected EV and chickenpox-infected VZV, we categorized the patients into four groups: EV infection, VZV infection, mixed infection and sequential infection with both viruses. A total of 85 patients were included: 33 with EV infection, 46 with VZV infection, 4 with mixed infection and 2 with a sequential infection involving both viruses (Figure 1A). Among the EV cases, CV-A6 was the predominant serotype, identified in 17 of the 33 patients (Figure 1B).

In our cohort, RT-PCR testing for SARS-CoV-2 (COVID-19) was performed in eight participants at the time of enrolment and all results were negative. No systematic screening for other viral or bacterial co-infection was undertaken. We recognise this as a limitation in interpreting co-infection status.

### Demographics and clinical presentations of the patients whose cases were difficult to distinguish between HFMD and chickenpox

Table 1 provides a comparative overview of the demographic data and clinical manifestations between the EV- and VZV-infected groups (Table 1). There was a greater percentage of males in both groups, with 63.64% in the EV group and 69.57% in the VZV group. The median age of patients in the EV group (31 months, IQR: 14–42) was lower than that of patients in the VZV group (72 months, IQR: 12–96), although this age difference was not statistically significant.

**Table 1:** Demographic characteristics and clinical manifestations at first visit of patients infected with EV or VZV virus.

Characteristic	Patients with EV infection (*N* = 33)	Patients with VZV infection (*N* = 46)	*P* value	OR	95% CI
Gender, *n* (%)			0.6323^a^	0.7656	0.2969**–**1.974
Male	21 (63.64)	32 (69.57)			
Female	12 (36.36)	14 (30.43)			
Male/Female	1.75	2.29			
Median age, month (P25, P75)	31.00 (14.00**–**42.00)	72.00 (12.00**–**96.00)	0.1179^b^		
Fever, *n* (%)	21 (63.64)	31 (67.39)	0.8116^a^	0.8468	0.3309**–**2.167
Median maximum temperature (P25, P75)	38.50 (38.00**–**39.40)	38.50 (38.15**–**38.95)	0.8007^b^		
Cough, *n* (%)	6 (18.18)	5 (10.87)	0.5116^a^	1.822	0.5054**–**6.570
Skin rash description, *n* (%)			0.6819^a^		
** **Macules and papules	32 (96.97)	38 (82.61)			
** **vesicles	23 (69.70)	26 (56.52)			
** **Ulceration	7 (21.21)	8 (17.39)			
Scabs	3 (9.09)	8 (17.39)			
Skin rash major site, *n* (%)			0.0227^a^^,^*		
Scalp and face	7 (22.58)	6 (17.14)	0.7579^a^	1.410	0.4174**–**4.762
Perioral	4 (12.90)	0 (0.00)	0.0437^a^^,^*	11.62	0.5997**–**225.1
** **Hands, feet	13 (41.94)	7 (20.00)	0.0648^a^	2.889	0.9683–8.619
** **Trunk	5 (16.13)	10 (28.57)	0.2561^a^	0.4808	0.1440–1.605
** **Limbs	12 (38.71)	5 (14.29)	0.0281^a^^,^*	3.789	1.152–12.47
Whole body skin	10 (32.26)	19 (54.29)	0.0866^a^	0.4010	0.1507–1.097
Unknown	2	11			
Skin rash pruritus, *n* (%)			0.0345^a^^,^*	0.1270	0.0211–0.7648
Yes	8 (57.14)	21 (91.30)			
No	6 (42.86)	2 (8.70)			
Unknown	19	23			
Oral or pharyngeal vesicles, *n* (%)			0.0238^a^^,^*	2.985	1.180–7.552
Yes	21 (63.64)	17 (36.96)			
No	12 (36.36)	29 (63.04)			

a Means Chi-square test or Fisher's exact test was used for statistical analysis.

b Means Mann Whitney *U* test was used for statistical analysis.

* Means *P* < 0.05 was considered significant. The cases with missing data marked as “Unknown” were excluded from the respective statistical analyses and the denominators for calculating percentages reflect only the number of patients with available information. OR, odds ratio; CI, confidence interval.

Fever was observed in 63.64% of patients with EV infection and 67.39% of patients with VZV infection, with median maximum temperatures of 38.50°C (IQR: 38.00–39.40) and 38.5°C (IQR: 38.15–38.95), respectively. Additionally, 18.18% of EV cases and 10.87% of VZV cases presented with cough. Skin rashes were not significantly different between the two groups. The skin rash types included macules and papules (96.97% in EV, 82.61% in VZV), vesicles (69.70% in EV, 56.52% in VZV), ulceration (21.21% in EV, 17.39% in VZV) and scabs (9.09% in EV, 17.39% in VZV). Pruritic rashes were reported in 57.14% of EV cases and 91.30% of VZV cases, a significant difference (*P* = 0.0345). Oral or pharyngeal vesicles were more common among EV patients (63.64%) than among VZV patients (36.96%, *P* = 0.0238). Notably, skin rash involvement was more widespread in VZV infection, affecting the whole body (54.29%), whereas skin lesions in patients with EV infection were more common in the limbs (38.71%, *P* = 0.0281) and perioral regions (12.90%, *P* = 0.0437).

### Hematological profiles of the hardly distinguishable cases between HFMD and chickenpox

As shown in Table 2, the hematological profiles overlapped significantly between the groups. It should be noted that laboratory data were available for a subset of patients (20/33 in the EV group and 27/46 in the VZV group) as complete blood count testing was not performed on all patients at the initial visit. Among the available data, neutrophil percentages, lymphocyte percentages and monocyte percentages did not differ significantly. Leukocyte count in the EV group was higher than that in the VZV group. However, atypical lymphocytes were detected in 25.93% of VZV cases but were absent in EV infection (*P* = 0.0154), suggesting a potentially significant marker for VZV.

**Table 2:** Laboratory results at first visit for patients with EV or VZV infection.

Characteristics	EV (*N* = 20)	VZV (*N* = 27)	*P* value	OR	95% CI
Median Leukocyte count (10^9^/L), (P25, P75)	8.15 (5.50–10.40)	6.00 (4.70–7.30)	0.0267^b^^,^*	–	–
Median Neutrophil (%), (P25, P75)	47.15 (37.00–59.95)	39.20 (31.00–51.00)	0.1058^b^	–	–
Median Lymphocyte (%), (P25, P75)	42.10 (26.30–46.68)	41.90 (29.70–58.00)	0.3125^b^	–	–
Median Monocyte (%), (P25, P75)	9.40 (6.43–11.58)	9.00 (7.00–12.60)	0.8520^b^	–	–
The detection rate of abnormal lymphocyte, *n* (%)	0 (0.00)	7 (25.93)	0.0154^a^^,^*	0.000	0.000–0.6959

a Means Fisher's exact test was used for statistical analysis.

b Means Mann Whitney *U* test was used for statistical analysis.

* Means *P* value was <0.05. OR, odds ratio; CI, confidence interval.

### Sequential and mixed infection patterns

Table 3 highlights infection patterns in patients with mixed and sequential infection. Among the six patients, two presented with anterior‒posterior sequential infection. A 4-year-old female first developed EV-associated symptoms (fever, rash on hands and feet, oral lesions). After the complete resolution of the initial EV infection, she subsequently developed VZV symptoms (pruritic trunk and facial rash) 19 days later. Similarly, a 14-month-old male initially presented with VZV. Following the full resolution of the varicella rash, he developed EV symptoms after 16 days.

**Table 3:** The clinical presentation at first visit of individual patients with sequential or mixed EV and VZV infection.

Patient No.	Age	Gender	Infection pattern	Virus Detection	Key Clinical Features at Presentation
1	4 y	F	Sequential	EV, then VZV (19 days later)	Initial (EV): Fever; vesicles on hands, feet and oral
					Subsequent (VZV): Fever; pruritic papules and vesicles on trunk and face
2	1y 2 m	M	Sequential	VZV, then EV (16 days later)	Initial (VZV): Pruritic vesicles on trunk and head
					Subsequent (EV): Fever; vesicles on hands, feet and oral
3	2y 4 m	F	Mixed	EV + VZV (simultaneous)	Fever; generalized rash with partial blistering; oral vesicles
4	10 y	M	Mixed	EV (CV-A6) + VZV (simultaneous)	Fever; widespread pruritic scattered rash and vesicles involving the entire body (including oral), with ulceration and scabbing
5	2y 8 m	F	Mixed	EV + VZV (simultaneous)	Fever; Scattered red papules all over the body, with scattered skin lesions on the hands, feet, and perianal area, some of which were accompanied by vesicles
6	15 y	M	Mixed	EV + VZV (simultaneous)	Fever; generalized pruritic vesicles covering the whole body, with partial scabbing

Both the 28-month-old female and the 15-year-old male who were infected with both EV and VZV presented with generalized rashes or vesicles on their bodies. A 10-year-old male with a CV-A6-positive EV presented with extensive rashes with ulceration and scabbing across the body. Another patient, a 32-month-old female, presented with rashes involving the entire body, including the perioral, hands, feet, and perianal regions, accompanied by vesicles and pruritus. These findings suggest that mixed infection lead to more widespread dermatological involvement.

## Discussion

Typical HFMD and varicella can often be distinguished by the distribution and characteristics of the rash, whereas atypical HFMD and chickenpox with similar clinical characteristics are often difficult to distinguish. Our findings analyzed the clinical characteristics, hematological profiles, and infection patterns of pediatric patients with cases of HFMD and chickenpox that were difficult to distinguish, providing valuable insights into their differentiation. Specifically, oral mucosal lesions and rashes localized to the limbs and perioral regions were more characteristic of EV infection at the patient's initial visit, whereas pruritic or widespread skin rashes were more indicative of VZV infection. Despite these distinctions, the overlap in clinical presentations, particularly in atypical cases, underscores the diagnostic challenges of relying solely on clinical symptoms for accurate diagnosis.

The laboratory results further highlighted the challenges in differentiating these clinically ambiguous HFMD and chickenpox cases. While neutrophil and lymphocyte percentages showed no significant difference between the EV and VZV groups, two distinct hematological patterns emerged. Patients with EV infection tended to present with a higher leukocyte count. This could reflect a stronger initial inflammatory response, commonly triggered by enterovirus. In contrast, Patients with VZV infection showed a key differentiator: atypical lymphocytes were detected in 25.93% of VZV cases but were completely absent in the EV group (*P* = 0.015). This finding aligns well with the known lymphotropism of VZV and the specific immune response it provokes, often leading to the appearance of these activated lymphocytes in the blood. Therefore, while total leukocyte count itself offers limited help in distinguishing these infections, the presence of atypical lymphocytes appears to be a more specific indicator pointing towards VZV in diagnostically uncertain scenarios.

Reports of patients co-infected with VZV and enterovirus were limited. Na's case mentioned that a 11-year-old girl who was infected with both VZV and enterovirus developed polymorphic rashes on her face and trunk, single-shaped painful pustules on her palms and soles, and painful ulcers in her mouth (9). Even in another case report, it was discovered that a 14-year-old male youth was simultaneously infected with the varicella-zoster virus and enterovirus, which caused meningitis (10). In our study, four cases of co-infection were identified. All these cases presented with extensive dermatological involvement, including vesicular rashes, ulceration, or scabbing across the body. These findings suggest that mixed infection may lead to more widespread dermatological involvement. It is important to note that the number of cases with mixed infection in our study was very small, which limits the statistical power and the generalizability of our conclusions. The small sample size increases the susceptibility to selection bias and makes it challenging to draw definitive conclusions about the prevalence or distinct clinical course of co-infection compared to single infection. Future studies with larger cohorts are necessary to validate these preliminary observations and to better understand the epidemiology and clinical impact of EV and VZV co-infection. Reports of sequential infection patterns were rare. Whether EV infected first or VZV infected first, the clinical symptoms presented were all related to the corresponding pathogen in our cases. It should be noted that patients with virus-virus co-infection or sequential infection may not be uncommon, as there were not sufficient widespread diagnostic tools available in the past for a complete diagnosis.

It is worth noting that although only 8 participants underwent RT-PCR testing for SARS-CoV-2 and all were negative, we cannot rule out subclinical or other viral/bacterial co-infection, which may modulate host-pathogen interactions. Prior vaccination against SARS-CoV-2 may shift baseline immunity and affect susceptibility or response to other infection (12), even if vaccination status was not uniformly recorded. Moreover, the rapid evolution of SARS-CoV-2 lineages across continents (13) underscores that variant-specific transmissibility or immune evasion could influence co-infection risk or immune-state dynamics. Persistent or long-term SARS-CoV-2 infection (14, 15) may leave residual immune dysfunction or viral reservoirs, thereby altering host responses to additional pathogens. Finally, diagnostic limitations, including low viral load infection, variant escape from standard assays, and incomplete screening of other pathogens (16), mean that undetected co-infections or persistent states might bias the interpretation of our findings. Together, these factors recommend cautious interpretation of causality in our results and support further studies with comprehensive pathogen screening, vaccination history, viral genome analysis, and longitudinal follow-up.

Notably, Coxsackievirus A6 (CV-A6) was predominant in the EV infection group and was detected in 51.52% of the patients. CV-A6 has been identified as a leading serotype in severe and atypical cases of HFMD (17, 18), often mimicking varicella-like presentations with extensive rashes involving the trunk and limbs (3). This overlap complicates the differentiation of HFMD from chickenpox, while atypical chickenpox presentations mimicking HFMD have also been reported (8). The emergence of CV-A6 as a predominant serotype in our study is consistent with recent studies showing that CV-A6 is more likely to cause severe dermatological manifestations and can overlap with symptoms observed in VZV infection (17, 18). This highlights the importance of including CV-A6-specific testing in the diagnostic workup for pediatric patients presenting with atypical HFMD, as it may lead to better differentiation from VZV-related conditions.

The findings of our study have significant implications for improving the diagnosis and management of pediatric EV and VZV infection, especially in regions where both viruses cocirculate. Advanced virological diagnostic methods, such as RT‒PCR and serotype-specific testing, are essential for accurate identification and differentiation. By providing a clearer understanding of the clinical and laboratory features distinguishing EV and VZV infection, as well as highlighting the potential severity of mixed and sequential infection, this research offers essential insights that could improve patient outcomes and treatment strategies.

However, our study has several limitations. Its retrospective design and relatively small sample size may have limited the detection of statistically significant differences between the two groups, particularly in terms of subtle clinical markers. Additionally, the study's focus on a pediatric cohort restricts the generalizability of the findings to adult populations or immunocompromised patients, who may exhibit different clinical or immune responses to EV and VZV infection. As a single-center study, our findings may be subject to selection bias and may not be fully generalizable to other populations or healthcare settings. The absence of follow-up data limits our ability to comment on the long-term outcomes and potential sequelae of these infection, particularly in cases of mixed or sequential infection. Furthermore, while we explored common clinical and laboratory markers, more in-depth investigations into inflammatory biomarkers or cytokine profiles could provide a deeper understanding of the pathophysiology of these infections.

Future studies should aim to include larger, multicenter cohorts with diverse age groups and immune statuses to increase the generalizability of the findings. Advanced diagnostic tools, such as next-generation sequencing, and a deeper exploration of immunological responses in coinfected and sequentially infected patients could significantly refine our understanding of these infections. Investigating the immunopathological mechanisms of EV and VZV interactions will be crucial for improving therapeutic strategies and managing severe cases more effectively.

In conclusion, this study highlights the challenges of distinguishing between EV and VZV infection on the basis of clinical presentation alone, particularly in cases of atypical HFDM, chickenpox or mixed infection. These findings emphasize the importance of early virological testing and serotype-specific diagnostics in improving diagnostic accuracy and informing treatment strategies. The identification of CV-A6 as a predominant serotype in atypical HFMD adds complexity to the clinical landscape, suggesting the need for further research into the full spectrum of EV serotypes and their clinical implications. On the other hand, it is hoped that this study can provide some guidance for the diagnosis of HFMD and chickenpox that are difficult to distinguish or have mixed infection in the absence of early virological testing.

## Data Availability

The original contributions presented in the study are included in the article/Supplementary Material, further inquiries can be directed to the corresponding authors.
